# An Instrumental Variable Probit Modeling of COVID-19 Vaccination Compliance in Malawi

**DOI:** 10.3390/ijerph182413129

**Published:** 2021-12-13

**Authors:** Abayomi Samuel Oyekale, Thonaeng Charity Maselwa

**Affiliations:** Department of Agricultural Economics and Extension, North-West University Mafikeng Campus, Mmabatho 2735, South Africa; thonaengmaselwa@gmail.com

**Keywords:** COVID-19, vaccine, compliance, stress, instrumental variable probit model

## Abstract

COVID-19 remains a pressing development concern in Malawi. The third wave of viral infection upsurge raised significant concerns on people’s compliance with preventive methods already introduced by the government, among which vaccination is notable. This study analysed the factors influencing COVID-19 vaccination compliance in Malawi. The data were the ninth round of the telephone-based survey that was conducted by Malawi National Statistical Office (NSO) in 2021. The data were analysed with Instrumental Variable Probit model. The results showed that awareness of COVID-19 vaccines arrival was very high (98.19%). Additionally, 11.59% and 60.71% were already vaccinated and planning to be vaccinated, respectively. The Probit regression results showed that age of household heads, need of medical services, being worried of contracting COVID-19 and wearing of masks increased the probability of vaccination compliance, while stress indicators, being employed and not worried at all of contracting COVID-19 reduced it. It was concluded that drastic behaviour change would be needed to address corona virus pandemic in Malawi. There is the need to ensure equity across different age groups in access to vaccines. Further, interventions to ensure proper assessment of an individual’s COVID-19 risk and address psychological and emotional stress that are associated with ongoing pandemic would enhance vaccination compliance.

## 1. Introduction

Although Malawi is currently among the African countries with very low COVID-19 positive cases and reported deaths, the recent spike of positive cases in ongoing third wave may be severely devastating, with perplexing socio-economic consequences. Assessing the vulnerability of Malawians to COVID-19 in the wake of recent viral spike beckons at several healthcare service delivery indicators. First, the primary health care (PHC) model that is being used in Malawi, though hypothetically excellent, suffers from implementation deficiency in the form of poor staffing, poor funding and inadequate medical supplies [1]. Secondly, the efficiency dichotomy in healthcare service delivery between Malawi’s rural and urban areas reemphasizes the magnitude of prevailing inequity and rural emergency unpreparedness should COVID-19 infections go beyond the borders of urban and peri-urban centers [2].

Therefore, uncontrolled COVID-19 infections in Malawi will obviously put significant pressure on the prospects of economic growth and health policy efficiency, given the magnitude of the projected impacts of lockdowns on the entire economy [3]. It was projected that COVID-19 will reduce Malawi’s economic growth to 1.0% and 2.8% in 2020 and 2021, respectively [4]. However, the African Development Bank [5] submitted that Malawi’s economic growth in 2020 was 1.7%, which was indisputably a remarkable decline from its 5.7% value in 2019.

Furthermore, recurring waves of the COVID-19 pandemic pose some grave consequences on poverty reduction strategy and economic reforms in Malawi. This is a pressing concern, going by the fact that the state of Malawian economy prior to the COVID-19 pandemic was characterized by high poverty incidence (51% in 2011), and a projected decline to 38% was expected in 2020 if consumption grows by 10% and inequality reduces to its 2004 level [6]. Inadvertently, these conditions are unachievable largely due to the COVID-19 pandemic. Moreover, the extent of economic damages to Malawian economy since the commencement of the pandemic may be deeper than ever imagined. This may also affect the ability of government to address other unprecedented health problems.

Moreover, the recent twist of COVID-19 infections is therefore worrisome given the discovery of SARS-CoV-2 Delta variant in Malawi [7]. More importantly, in comparison with the Alpha variant, Callaway [7] noted that the Delta variant is 60% more transmissible and infected people are 50% more likely to be hospitalized [7]. Healthcare practitioners are wary of this viral strain given its distinguishable infection severity and hospitalization requirements. The Malawian government and several international stakeholders in the health sector are particularly worried about the recent development, given the Malawian’s historical record of lockdown resistance and generally low compliance with prescribed safety procedures [8,9].

Understanding the determinants of compliance with COVID-19 preventive guidelines is of significant relevance in addressing current and future infections in Malawi. This research is indisputably relevant to advancement of existing knowledge on vaccine hesitancy and promotion of efficiency in the ongoing efforts towards attainment of herd immunity against COVID-19 [10]. Although the conventional approaches of social distancing, crowd avoidance, mask wearing, hand washing and sanitization (among others) are effective preventive methods [11,12,13,14,15], boosting of the immune systems through vaccination is considered as a superior approach with lasting impacts [16].

In like manner, the peculiarity of the COVID-19 pandemic demands mass vaccination in order to attain herd immunity [17,18]. The decision to take COVID-19 vaccines, despite the understanding of the side effects, depends on several factors. Notable among these is an assessment of whether the benefits outweigh the risks. From economics point of view, however, attainment of a socially optimum level of vaccination is always difficult due to human tendency to free-ride [18]. This is even made worse in the present case since the coronavirus is progressively mutating into new variants, thereby compromising the immune systems of those already infected or vaccinated [19,20].

Moreover, health policy makers need to be well informed on vaccines’ hesitancy rates and the factors that would determine their acceptability. In some previous studies, 91.3% of elderly people in the United States indicated willingness to get COVID-19 vaccines [21]. In addition, safety perception, vaccine efficacy, race and gender influenced the decision to get vaccinated. In a study that was conducted in Bangladesh [22], the willingness to take a COVID-19 vaccine was 74.6% if the vaccine was freely administered, safe and effective. However, the results further showed that a minimum introduction of vaccination fees would reduce the willingness to be vaccinated to 46.5%, while age, sector of residence and the level of confidence in the healthcare system significantly influenced vaccination decision. In a study in the Mainland China [23], 77.4% of the respondents were willing to be vaccinated and 81.1% would be willing to pay. It was also found that the willingness to get vaccines was associated with education, while the acceptable price range for vaccines was US$75 to US$149. In another study, vaccination fees also reduced willingness to be vaccinated [24].

This study seeks add to the existing body of knowledge by extending the scope and analytical rigours of previous studies. Specifically, it seeks to determine the factors influencing COVID-19 vaccination compliant in Malawi. The estimated models also took cognizance of the endogeneity tendency of some of the variables, in order to ensure consistency of the estimated parameters. In the remaining parts of this paper, the methods of data analyses, results, discussion of findings and conclusion were presented.

## 2. Materials and Methods

### 2.1. The Study Area

Malawi is a landlocked country that shares its borders with Tanzania, Zambia and Mozambique in the southern part of Africa [4]. Reckoned as one of the poorest countries in the world, 76.36% of the labour force was engaged in agriculture in 2019 [25], with the majority practicing subsistence farming. Although recent economic reforms and structural changes are producing some positive results, poverty and inequality in Malawi are still perplexingly high. Life expectancy at birth increased from 51.7 years in 2009 to 63.7 years in 2019, while maternal fertility declined from 5.9 in 2009 to 4.3 in 2019. Infant mortality rate (per 1000 live births) was 66 in 2010 but declined to 35.3 in 2018 [26]. Malawi is also facing some pressing challenges from some non-communicable diseases. The healthcare facilities are largely owned by the government (63%) and Christian Association of Malawi (26%) [27].

### 2.2. The Data and Sampling Methods

The data were obtained from telephone based surveys that were conducted by Malawi’s National Statistical Office (NSO) with technical assistance from the World Bank [28]. The survey used the sampling frame of the Integrated Household Panel Survey (IHPS) that was developed in 2019. This framework is representative of the whole country with rural and urban households as the main sampling units. The households are groups of people who share their resource endowments for the maximization of their utilities. Since COVID-19 preventive protocols demand social distancing and some restrictions in people’s movements, the survey was implemented with phone calls. The survey was made possible because the sampling frame database of the 2019 IHPS contains 3181 households, among which 2337 provided their phone numbers or some reference numbers. The survey aimed to interview all 2337 households during the first Round but only 1729 households completed the survey. During the second Round, 1646 successfully completed the survey. The third, fourth and fifth Rounds had 1624, 1616 and 1589 households, respectively. During the sixth, seventh, eighth and ninth Rounds, 1592, 1560, 1551 and 1545 households completed the survey. This study used the ninth Round because it contains information on COVID-19 vaccination [28].

The ninth panel data were collected between 7 April and 23 April 2021. The respondents were the members of the selected households with proper knowledge of households’ activities. The data were collected by trained enumerators using phone tablets. The respondents were also expected to give verbal consents in order to show their willingness to participate in the surveys. Moreover, a compensation of 1000 Malawi Kwacha was given to every respondent as a way of appreciating their participation. The interview was conducted via Computer Assisted Telephone Interview (CATI) and data were captured with Survey Solutions software that was developed by the World Bank [28].

### 2.3. Limitations of the Data

The dataset that was used for this study was collected with telephone interviews. The selection of the sampling units by focusing on those households that provided their phone numbers during the 2019 IHPS may have compromised the representativeness of the survey. Similarly, the dataset also suffered reduction in sample size due to drop-out of some respondents as the surveys progressed. This is a peculiar problem in panel data surveys. In addition, the approach of this study is different from some qualitative assessment of vaccine hesitancy that had used the VAX-scale questionnaire [29]. This is because the intention of this study was to understand the correlates of individuals’ willingness to be vaccinated in line with some previous studies [21,22,23,24].

### 2.4. Data Merging and Variable Construction

The coded dataset was presented in three formats which are SPSS, STATA and CSV. For this study, the SPSS datafile format was used. This folder contains some separate sections of the questionnaire for all the Rounds of survey that had been completed. The data files for Round 9 were merged based on households’ identification codes. However, Section 2 of the dataset was the households’ roster, for which 7944 respondents were coded. These data points exceed the 1545 data points that were had for the households. In order to include household heads’ age and gender, the data in Section 2 of the questionnaire were filtered and merged with the main household files using households’ identification codes. In addition, some households did not indicate their actual ages, but the oldest persons were selected as the heads of those households.

Furthermore, two variables representing stress and food problem indicators were generated in the course of data analysis using principal component analysis (PCA). Aggregating these responses into composite indices to represent indicators of health and food problems addresses the multicollinearity problem that would have arisen if they were estimated as dummy variables. This is a result of a high level of correlation that would exist between some of the variables. The indicator of stress was constructed from a series of symptoms that the respondents may have recently observed as contained in Section 4b of the questionnaire. Those questions, for which yes or no answers were required, are:

- little interest or pleasure in doing things;
- feeling down, depressed, or hopeless;
- trouble falling or staying asleep, or sleeping too much;
- feeling tired or having little energy;
- poor appetite or overeating;
- feeling bad about yourself/or that you are a failure/have let yourself or family down;
- trouble concentrating on things, such as reading the newspaper/watching TV; and
- moving or speaking so slowly/or fast that other people could have noticed.

Furthermore, food problem index was computed from the responses to the questions which are:

- household worried about not having enough food to eat;
- household unable to eat healthy and nutritious/preferred foods;
- household ate only a few kinds of foods;
- household had to skip a meal;
- household ate less than you thought you should;
- household ran out of food;
- household hungry but did not eat; and
- household went without eating for a whole day

### 2.5. Estimated Models

The Instrumental Variable Probit regression model was used for data analysis. This model is preferred if one or more independent variables are endogenous [30]. Estimating this model requires the formulation of the Probit regression model which would have inconsistent parameter if the suspected endogeneity problem is not properly addressed. A standard Probit regression model can be stated as follows:

(1) $$Prob\left(V_{i}=1/X\right)=\int_{-\infty }^{X_{i}\beta }{\left(2\pi \right)}^{-1/2}\exp\left(-\frac{t^{2}}{2}\right)dt=\phi \left(X_{i}\beta \right)$$

In Equation (1), ϕ is the cumulative distribution function of a standard normal variable. The operation of this function, which is an advantage over the linear probability model is that estimated probabilities (*p*_*i*_) complies with the condition, 0 ≤ *p*_*i*_ ≤ 1. Additionally, *X* represents the vector of explanatory variables and 𝛽 is a vector of the parameters of the explanatory variables. Equation (1) can be restated with introduction of an endogenous regressor and presented in a reduced form as:

(2) $$V_{i}=\alpha +\overset{k}{\underset{i}{\sum }}\beta_{i}X_{i}+\gamma H_{i}+e_{i}$$

The model for those willing to be vaccinated, which in this estimation also included those who had been vaccinated in order to retain the degree of freedom and to ensure complete capturing of vaccination compliance, is stated as:

(3) $$VC_{i}=\omega +\overset{k}{\underset{i}{\sum }}\pi_{i}X_{i}+\varphi H_{i}+n_{i}$$

In Equation (2), *V_i_* is COVID-19 vaccination status (yes = 1, 0 otherwise), $X_{i}$ is a vector of explanatory variables, and *H*_i_ is stress indicator that was computed with PCA. In addition, $\alpha ,\;\;\beta_{i}$ and γ are the estimated parameters. In Equation (3), $VC_{i}$ denotes COVID-19 vaccine compliance coded as 1 for those who were either vaccinated or willing to be vaccinated and 0 otherwise. Further, ω, $\pi_{i}$ and φ are estimated parameters and $e_{i}$ and $n_{i}$ are the error terms. If *Cov* ($e_{i},$*H*_i_) = 0 or *Cov* ($n_{i},$*H*_i_) = 0, then there is no endogeneity problem. However, it is expected that these conditions may not hold because the dependent variables ($V_{i}$ and $VC_{i}$) and the suspected endogenous variable $(H_{i}$) are health variables that may share the same explanatory variables which had been omitted from Equations (2) and (3). A good example is a situation where an individual suffers from allergic reactions from some medications or foods which may affect the decision to be vaccinated and the person’s health. This is a critical point because there have been several claims on the side effects of COVID-19 vaccines and previous studies have highlighted the issue of safety as a fundamental factor influencing decision to get vaccinated [20,21,22,23,24]. This implies that endogeneity is a potential problem in Equations (2) and (3). Therefore, instrumental variable(s) must be engaged in the specification of the vaccination compliance model. The stress equation is represented as Equation (4):

(4) $$H_{i}=\delta +\overset{k}{\underset{i}{\sum }}\partial_{i}X_{i}+\mu F_{i}+v_{i}$$

In order to correct the endogeneity problem, Equation (2) was restated as:

(5) $$V_{i}=\alpha +\overset{k}{\underset{i}{\sum }}\beta_{i}X_{i}+\gamma H_{i}+\tau v_{i}+e_{i}$$

and Equation (3) was restated as:

(6) $$VC_{i}=\omega +\overset{k}{\underset{i}{\sum }}\pi_{i}X_{i}+\varphi H_{i}+\sigma v_{i}+n_{i}$$

The variable $F_{i}$ in Equation (4) is a food problem index, which was computed with PCA. This is the instrumental variable for *H*_i_. Although instrument selection is a major hurdle in estimating models with endogenous regressors, one critical rule of thumb applies. This is the fact that the selected instrument(s) must be correlated with the endogenous regressor (*H*_i_.) but not correlated with the dependent variables in Equations (5) and (6). The explanatory variables for Equations (2)–(4) are stress index, gender of household head (male = 1, 0 otherwise), age of the household head, hand washing (yes = 1, 0 otherwise), mask wearing (yes = 1, 0 otherwise), medical services needed (yes = 1, 0 otherwise), worried family contract COVID (yes = 1, 0 otherwise), not too worried family contract COVID (yes = 1, 0 otherwise), not worried at all that family contract COVID (yes = 1, 0 otherwise), finance moderately threatened by COVID-19 (yes = 1, 0 otherwise), finance not much threatened by COVID-19, finance not threatened at all by COVID-19 (yes = 1, 0 otherwise) and employment status during last survey (employed = 1 and 0 otherwise).

STATA 17 software was used for data analysis and it generated the statistics for Wald’s test of exogeneity. If this parameter is not statistically significant, the null hypothesis of exogeneity is to be accepted. This also implies that the parameters of the residuals from Equation (4) (τ and σ) in Equations (5) and (6) are not statistically significant (*p* > 0.05). However, if it is statistically significant, the null hypothesis of exogeneity should be rejected. This then implies that $H_{i}$ is truly endogenous, and estimating the model with standard Probit regression model would produce inconsistent parameters.

## 3. Results

### 3.1. Respondents’ Demographic Characteristics and Decision to Be Vaccinated

**Figure 1:**
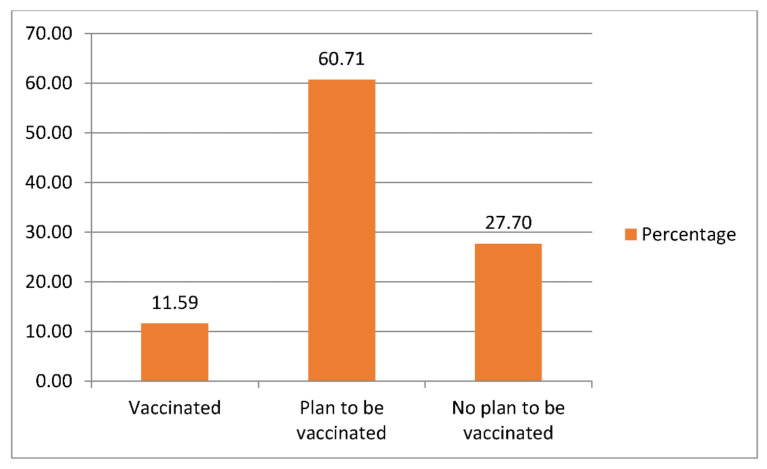
Distribution of the respondents based on their vaccination status.

Figure 1 shows the percentage distribution of the respondents based on being vaccinated (11.59%), planning to be vaccinated (60.71%) and not willing to be vaccinated (27.70%). Table 1 also shows the distribution of the respondents’ socioeconomic characteristics across their COVID-19 vaccination status. The results show that 78.90% of the respondents were males. However, 75.98% and 80.38% of the vaccinated and those planning to be vaccinated were males. Male respondents constituted 76.87% of those who were not planning to be vaccinated. Awareness of COVID-19 vaccine’s arrival in Malawi was high (98.19%). Specifically, 6.54% of those who were not planning to be vaccinated claimed not to be aware of arrival of vaccines. This proportion is the highest when compared to the other groups of respondents. The age distribution of the respondents shows that 27.96% of all the respondents belonged to age group 35 < 45 years. This age group also constitutes the highest proportion (25.70%) of those who were vaccinated. Medical services were not required by 57.61% of all the respondents in the previous four weeks prior to the survey. In addition, 71.26% of all the respondents worked since the previous interview. This can be compared to 81.42% that indicated to have worked or conducted some business in a week prior to the current interview.

Table 2 further shows the distribution of the respondents’ vaccination status across their compliance with safety guidelines and perceptions of their households’ vulnerability to COVID-19. Washing of hands after being in public places was adopted by majority of the respondents (91.00%). A similar finding applies to wearing of face masks in public places (89.23%). The table further shows that 70.16% of all the respondents were very worried of contracting COVD-19. Further, 59.22% of the respondents who were vaccinated were very worried about contracting the disease. However, those who were not worried at all form 10.06% of those who were vaccinated. The results further show that across all the groups of the respondents, majority of them perceived COVID-19 as substantial threat to their households’ finances. Specifically, about 85% of all the respondents indicated that COVID-19 is a substantial or moderate threat to their finances.

Table 3 shows the distribution of the respondents across the different forms of stress that were experienced. It reveals that 27.77% of all the respondents had little interest or pleasure in doing things. It also shows that 20.67% of the vaccinated respondents had little interest or pleasure in doing things. This is the lowest percentage across the different groups. In addition, feeling down, depressed or hopeless was reported by one-third of all the respondents. The respondents that were vaccinated also had the lowest percentage for this experience. Experiences of trouble falling or staying asleep, or sleeping too much, were reported by about one-quarter of all the respondents, while feeling tired or having little energy was reported by 33.27%. Poor appetite or overeating was reported by 18.12% of the respondents, while feeling bad about oneself was reported by 32.23%. Additionally, 16.12% of all the respondents had trouble concentrating on some things, such as reading the newspaper or watching TV. Moving or speaking so slowly/or fast that other people could have noticed was experienced by 14.11%.

### 3.2. Determinants of Being Vaccinated and Vaccination Compliance

Table 4 shows the results of the estimated models being vaccinated and being vaccine compliant (vaccinated and planning to be vaccinated). The results showed that stress index variable was truly endogenous in the two models. This conclusion was reached because the computed Wald test statistics are statistically significant (*p* < 0.05). The implication is that estimating the models using a standard Probit model would produce inconsistent parameters. The model also produced good fits for the data. This is reflected by statistical significance of the computed Wald Chi Square statistics (*p* < 0.05). This also shows that the estimated parameters are not jointly equal to zero. Therefore, the included variables have some influences on vaccine compliance.

Among the variables that were included, stress index shows statistical significance (*p* < 0.01) in Model 1. The parameter for Model 2 was only significant at the 10% level. However, there is consistency in the sign of the parameters in the two models. The results show that as stress index increased, the probability of being vaccinated or vaccination compliant decreased. Gender did not show statistical significance in the two models (*p* > 0.10) and there is no consistency in the sign of the parameters. Age of household heads showed statistical significance (*p* < 0.01) in the two models and with positive sign. These results show that as age increased, the probability of being COVID-19 vaccinated or vaccination compliance increased. In Model 1, the parameter of medical service needed is with positive sign and statistically significant (*p* < 0.01). This shows that those who needed medical services in previous four weeks before the survey had significantly higher probability of being vaccinated. Similarly, the variable employed during last survey in Model 1 is statistically significant (*p* < 0.01). This shows that those who were working during previous survey had lower probability of being vaccinated.

Furthermore, out of the variables that were included to capture risk perception, none of the perception of COVID-19 as threat to finance show statistical significance (*p* > 0.10). However, among the variables that captured being worried of contracting COVID-19 in Model 1, the parameters of worried family contracts COVID and not too worried family contracts COVID are statistically significant at 10% and 5% levels respectively. These results show that compared to those respondents who were very worried about contracting COVID-19, being worried or not too worried increased the probabilities of being vaccinated. However, in Model 2, the parameter of not worried at all of family member contracting COVID-19 shows statistical significance (*p* < 0.05). The result shows that compared to those who were very worried of contracting the disease, not being worried at all of family members contracting COVID-19 reduced the probability of being vaccination compliance. Finally, in Models 1 and 2, out of the protective behaviour variables, only wearing of mask parameters are statistically significant (*p* < 0.05). These results show that those respondents who were wearing masks whenever they were among crowds of people had significantly higher probabilities of being vaccinated or vaccination compliance.

## 4. Discussion

Most of the respondents were aware of the arrival of COVID-19 vaccines in Malawi. This is a reflection of effectiveness of different media being engaged by the Malawian government to promote access to information on COVID-19 irrespective their places of residence [31]. COVID-19 vaccination compliance in Malawi is fairly good with 11.59% of the respondents already vaccinated and 60.71% planning to be vaccinated. However, in the contexts of several criticisms that the Theory of Planned Behaviour had suffered, willingness to carry out a behavioural obligation may not always transpire into actual action due to several logistical, psychological and economic constraints [32]. In the context of this study, people who are willing to be vaccinated may not be able to do so as a result of shortage of vaccines and geographical locations. More importantly, the ultimate goal of vaccination may be completely defeated if the population does not attain herd immunity [33]. Therefore, under current pandemic, the requisite vaccination coverage in order to attain herd immunity is not known. However, when gauged along with diseases such as measles and polio with herd immunity attainment of 95% and 80% vaccination coverage, respectively [33], one would understand that the fight against COVID-19 may not have begun.

Gender of the respondents did not significantly influence COVID-19 vaccination compliance. This is contrary to some previous studies. Specifically, Green et al. [34] found women to have lower willingness to participate in COVID-19 trial vaccines. A similar finding was reported by Karisson et al. [35]. In a study by Galasso et al. [36], women perceived greater health risks from COVID-19 than men, while a contrary finding was reported by Griffith et al. [37].

Age is always an important factor in assessing the risk factors and vulnerability of individuals to some diseases. The case of COVID-19 is not an exception because the virus disproportionately produces more severe illness and death among aged persons than youths. The results show that age significantly influences compliance with COVID-19 vaccination. This finding is in accordance with the findings in some previous studies [22,29,35,38]. Age is a measure of objective risk and vulnerability to COVID-19 [35], and it is a measure of general decline in the immune system as people grow old [39,40,41]. The results also show that those who required medical services four weeks prior to the survey had a significantly higher probability of being vaccinated. Although some other household members may require medical services, households with aged heads may likely require more medical services [42].

Compliance with COVID-19 prevention through hand washing after being to public places and wearing of masks in public places was quite high. The results further show that wearing of masks significantly enhanced COVID-19 vaccination compliance. This in line with the findings of Kao et al. [9] that less costly preventive measure can be easily adopted by individuals against disease infection. Another study by Drobnik et al. [43] emphasized the role of face masks in preventing transmission of the coronavirus among healthcare workers. This is a very vital point in Malawi’s context, being one of the poorest countries in the world, with the majority of people depending on daily informal businesses and farm activities. The reflection of cost-benefit assessment in individuals’ engagement in disease prevention underscores some economic senses and rationality [44]. It can also explain the finding of the study that those employed during the previous survey had a lower probability of being vaccinated. Vaccination would require setting time aside from daily busy schedules and the queue may be long at its commencement in Malawi [31].

Perception of health risk is a major factor influencing decisions on healthcare treatment seeking behaviour and compliance with preventative behaviour [35,45]. The findings showed that not being worried of contracting COVID-19 increased the likelihood of getting vaccinated or planning to get the jabs. This is in accordance with some previous studies that had emphasized association between compliance with preventive behaviour and health risk perception probably due to their age or presence of predisposing health problems such as cancer, HIV or any non-communicable disease [35]. Perceived risks are sometimes measured based on an individual’s assessment of the probability of contracting the disease, severity of the symptoms, treatment procedures and what would be the psychological and emotional disturbances of caregivers [46,47,48,49,50,51]. It was also found that respondents with high levels of stress had not been vaccinated. This result may be a reflection of psychological disturbances that are associated with ongoing pandemic given some uncertainties surrounding individuals’ safety. Therefore, those who had been vaccinated may have lower feelings of anxiety and fear of COVID-19 that can substantially reduce their level of stress and promote their health status [52,53].

## 5. Conclusions

Attainment of success in addressing COVID-19 is a top development agenda among health policy makers and stakeholders across the world. This is, however, becoming more difficult due to the intermittent resurgence of viral infections in some countries. In Malawi, a recent resurgence of COVID-19 infections portends some economic consequences on the fragile economy, if there would the need for complete economic lockdown. This is so, given the failure of government to implement a successful lockdown in the wake of the virus’ transmission in 2020. The intention of international policy makers is to provide some workable mechanisms through vaccination for enhancing the immune system. The Malawian government is freely supplying vaccines to citizens and some citizens have been vaccinated against COVID-19. This study therefore examined the factors explaining access to vaccines and willingness to be vaccinated against the disease.

The findings have highlighted high awareness of arrival of COVID-19 vaccines. The findings have some policy implications. Specifically, vaccination compliance was promoted by age of the respondents. This is in line with the assumption that aged people are most vulnerable to COVID-19. This can be misleading given that in some countries, youths have been major sources of virus transmission. It is therefore important to explore COVID-19 prevention with some sense of equity in administration of preventive vaccines because the youths may not readily show symptoms, but can act as sources of viral transmission. In addition, perception of health risk associated with COVID-19 is a fundamental factor promoting compliance with vaccination. This therefore buttresses the need for interventions to assist individuals to properly evaluate their vulnerability levels in order to ensure proper compliance with vaccination as an effective preventative method.

Although administration of COVID-19 vaccines may take cognizance of health status of individuals, especially those with pressing health problems that often aggravate the severity of infection, some psychological issues that culminate in high stress levels can have devastating consequences on people’s well-being during an ongoing pandemic. It is therefore important for medical services to go beyond administration of medications. There is the need to cater for effective procedures to address mental health and psychological distresses that may have resulted from the pandemic through counseling and other forms of psychological and emotional support mechanisms.

## Figures and Tables

**Table 1:** Vaccination Status and Respondents’ Demographic Characteristics.

Variables	Vaccinated (*n* = 179)		Planning to Be Vaccinated (*n* = 938)		Not Planning to Be Vaccinated (*n* = 428)		All Respondents (*n* = 1545)	
	Freq	% of Total	Freq	% of Total	Freq	% of Total	Freq	% of Total
Gender								
Female	43	24.02	184	19.62	99	23.13	326	21.10
Male	136	75.98	754	80.38	329	76.87	1219	78.90
Aware of vaccine								
Now Aware	0	0.00	0	0.00	28	6.54	28	1.81
Aware	179	100.00	938	100.00	400	93.46	1517	98.19
Age groups								
<25	7	3.91	75	8.00	52	12.15	134	8.67
25 < 35	34	18.99	244	26.01	132	30.84	410	26.54
35 < 45	46	25.70	276	29.42	110	25.70	432	27.96
445 < 55	39	21.79	160	17.06	63	14.72	262	16.96
55 < 65	31	17.32	110	11.73	41	9.58	182	11.78
65 and above	22	12.29	73	7.78	30	7.01	125	8.09
Medical services needed								
No	100	55.87	542	57.78	248	57.94	890	57.61
Yes	79	44.13	396	42.22	180	42.06	655	42.39
Worked last week								
No	48	26.82	160	17.06	79	18.46	287	18.58
Yes	131	73.18	778	82.94	349	81.54	1258	81.42
Worked during last survey								
No	68	37.99	259	27.61	117	27.34	444	28.74
Yes	111	62.01	679	72.39	311	72.66	1101	71.26

**Table 2:** Respondents’ Perceptions of Vulnerability to COVID-19, Safety Compliance and Vaccination Status.

Variables	Vaccinated (*n* = 179)		Planning to Be Vaccinated (*n* = 938)		Not Planning to Be Vaccinated (*n* = 428)		All Respondents (*n* = 1545)	
	Freq	% of Total	Freq	% of Total	Freq	% of Total	Freq	% of Total
Hand washing								
No hand washing or did not go out	14	7.82	69	7.36	56	13.08	139	9.00
Washed hands	165	92.18	869	92.64	372	86.92	1406	91.00
Mask wearing								
No mask wearing or did not go out	11	6.15	88	9.38	66	15.42	165	10.68
Wore masks	168	93.85	850	90.62	362	84.58	1380	89.32
COVID-19 and Health								
Very worried of having COVID-19	106	59.22	683	81.50	295	68.93	1084	70.16
Somewhat worried of having COVID-19	28	15.64	110	13.13	36	8.41	174	11.26
Not too worried of having COVID	27	15.08	75	8.95	43	10.05	145	9.39
Not worried at all of having COVID	18	10.06	70	8.35	54	12.62	142	9.19
COVID-19 and Finance								
COVID-19 is substantial threat to finance	112	62.57	678	80.91	300	70.09	1090	70.55
COVID-19 is moderate threat to finance	29	16.20	132	15.75	61	14.25	222	14.37
COVID-19 is not much threat to finance	26	14.53	86	10.26	48	11.21	160	10.36
COVID-19 is not threat at all to finance	12	6.70	42	5.01	19	4.44	73	4.72

**Table 3:** Respondents’ Experiences of Stress Across Their Vaccination Status.

Variables	Vaccinated (*n* = 179)		Planning to Be Vaccinated (*n* = 938)		Not Planning to Be Vaccinated (*n* = 428)		All Respondents (*n* = 1545)	
COVID-19 and Health	Freq	% of Total	Freq	% of Total	Freq	% of Total	Freq	% of Total
Little interest or pleasure in doing things								
No	142	79.33	656	78.28	318	74.30	1116	72.23
Yes	37	20.67	282	33.65	110	25.70	429	27.77
Feeling down, depressed, or hopeless								
No	126	70.39	615	73.39	289	67.52	1030	66.67
Yes	53	29.61	323	38.54	139	32.48	515	33.33
Trouble falling or staying asleep, or sleeping too much								
No	132	73.74	684	81.62	340	79.44	1156	74.82
Yes	47	26.26	254	30.31	88	20.56	389	25.18
Feeling tired or having little energy								
No	129	72.07	622	74.22	280	65.42	1031	66.73
Yes	50	27.93	316	37.71	148	34.58	514	33.27
Poor appetite or overeating								
No	147	82.12	761	90.81	357	83.41	1265	81.88
Yes	32	17.88	177	21.12	71	16.59	280	18.12
Feeling bad about yourself/or that you’re a failure/have let yourself or family								
No	134	74.86	630	75.18	283	66.12	1047	67.77
Yes	45	25.14	308	36.75	145	33.88	498	32.23
Trouble concentrating on things, such as reading the newspaper/watching TV								
No	145	81.01	786	93.79	365	85.28	1296	83.88
Yes	34	18.99	152	18.14	63	14.72	249	16.12
Moving or speaking so slowly/or fast that other people could have noticed?								
No	152	84.92	795	94.87	380	88.79	1327	85.89
Yes	27	15.08	143	17.06	48	11.21	218	14.11

**Table 4:** Instrumental Variable (IV) Probit Result of the Determinants of COVID-19 Vaccination Compliance.

Variables	Vaccinated (Model 1)			Positive Vaccine Intention and Vaccinated (Model 2)		
	Coefficient	Std. Err.	Z Stat	Coefficient	Std. Err.	Z Stat
Demographic/health						
Stress index	−0.3117178 ***	0.0535994	−5.82	−0.1020463 *	0.058470	−1.75
Gender of household head	−0.0564463	0.0961462	−0.59	0.1172023	0.083045	1.41
Age of the household head	0.011972 ***	0.0028735	4.17	0.0087886 ***	0.002505	3.51
Medical services needed	0.3180021 ***	0.0908427	3.50	0.0887663	0.0836925	1.06
Employed during last survey	−0.2675059 ***	0.0850981	−3.14	−0.0880712	0.0769721	−1.14
Risk perception						
Worried family contracts COVID	0.2285566 *	0.1256379	1.82	0.1960976 *	0.1197572	1.64
Not too worried family contracts COVID	0.3229509 **	0.133186	2.42	−0.0891405	0.1229729	−0.72
Not worried at all family contracts COVID	0.0659449	0.1467924	0.45	−0.3235615 **	0.1237355	−2.61
Finance moderately threatened by COVID	−0.135423	0.120778	−1.12	−0.0751409	0.1060315	−0.71
Finance not much threatened by COVID	−0.0281268	0.1361904	−0.21	−0.0878501	0.1236292	−0.71
Finance not threatened at all by COVID	0.0995716	0.1846589	0.54	0.1537056	0.173889	0.88
Protective behaviour						
Hand washing	−0.2759615	0.1826417	−1.51	0.1598855	0.1428438	1.12
Mask wearing	0.450405 **	0.1896148	2.38	0.2829097 **	0.1314992	2.15
Constant	−1.701197 ***	0.2460611	−6.91	−0.2144785	0.179874	−1.19
Diagnostic indicators						
Athrho	0.5450511 ***	0.1170509	4.66	0.235604 **	0.1015182	2.32
Lnsigma	0.4562054 ***	0.0179896	25.36	0.4562053 ***	0.0179896	25.36
Rho	0.4968019	0.0881613		0.2313392	0.0960852	
Sigma	1.578074	0.0283889		1.578074	0.0283889	
Number of obs	1545			1545		
Wald Chi Square (13)	98.03 ***			46.87 ***		
Log likelihood	−3416.39			−3783.771		
Wald test of exogeneity	21.68 ***			5.39 **		
VIF	1.18			1.18		

***–Significant at 1% level, **–Significant at 5% level; *–Significant at 10% level.

## Data Availability

The dataset can be downloaded from www.microdata.worldbank.org. The author was granted the permission to use the dataset but not authorized to share the data with any third party.
